# Stereotyped whistles in southern resident killer whales

**DOI:** 10.7717/peerj.12085

**Published:** 2021-08-27

**Authors:** Marie Souhaut, Monika W. Shields

**Affiliations:** 1 Orca Behavior Institute, Friday Harbor, WA, USA; 2 Marine and Freshwater Research Centre, Galway-Mayo Institute of Technology, Galway, Ireland

**Keywords:** Killer whales, Animal communication, Vocal dialects, *Orcinus orca*, Acoustic communication, Cetaceans

## Abstract

The endangered Southern Resident killer whales (*Orcinus orca*) of the northeast Pacific region use two main types of vocal signals to communicate: discrete calls and whistles. Despite being one of the most-studied cetacean populations in the world, whistles have not been as heavily analyzed due to their relatively low occurrence compared to discrete calls. The aim of the current study is to further investigate the whistle repertoire and characteristics of the Southern Resident killer whale population. Acoustic data were collected between 2006–2007 and 2015–2017 in the waters around San Juan Island, Washington State, USA from boats and from shore. A total of 228 whistles were extracted and analyzed with 53.5% of them found to be stereotyped. Three of the four stereotyped whistles identified by a previous study using recordings from 1979–1982 were still occurring, demonstrating that whistles are stable vocalizations for a period of more than 35 years. The presence of three new stereotyped whistles was also documented. These results demonstrate that whistles share the longevity and vocal tradition of discrete calls, and warrant further study as a key element of Southern Resident killer whale communication and cultural transmission.

## Introduction

Studying acoustic communication in cetaceans provides insights not only into their behavior, but, given the socially-learned vocal traditions present in many species, also into their culture. By classifying vocal signals into categories and assessing usage by different populations, groups, or individuals, it is possible to gain insights into the usage, function, and importance of different signal categories (Deecke, Ford & Spong, 1999). Odontocetes, or toothed whales, are a subset of the cetaceans known for their large brains and complex social structure and often have correspondingly complex vocal communication systems (Marino et al., 2007). These communication systems are particularly well-studied among species such as sperm whales (*Physeter macrocephalus*, *e.g.*, Cantor et al., 2019), bottlenose dolphins (*Tursiops truncatus*, *e.g.*, Janik & Sayigh, 2013) and killer whales (*Orcinus orca*, *e.g.*, Ford, 1989).

The sounds produced by killer whales have been classified into three main categories: clicks, pulsed calls, and whistles. Clicks are very short, broadband signals used in echolocation, the main function of which is navigation. Pulsed calls are structurally variable signals with distinct tonal qualities and harmonic structure. These calls, typically 0.5–1.5 s in duration, are the primary social vocalization of killer whales, and have been further categorized into discrete, variable, and aberrant calls (Ford, 1987). Discrete calls, also referred to as stereotyped calls, are the most common acoustic signal produced by killer whales and tend to have a distinct enough structure that they are easily distinguishable by the human ear. Variable calls are non-repeated pulsed vocalizations that are rare (Rehn, Teichert & Thomsen, 2007) while aberrant calls are alternative or distorted versions of discrete calls (Ford, 1989). In killer whale populations in the northeast Pacific, discrete calls are known to be stable over more than 50 years, meaning that their tonal structure is the same in both modern recordings and historic recordings (Ford, 1987; Wieland, Jones & Renn, 2010). Discrete call repertoires form the basis of dialects shared among different killer whale social groups, from matriline to pod to clan.

Whistles are non-pulsed continuous signals with much simpler harmonic structure. They can be less than a second to more than 10 s in duration (Ford, 1987). Whereas whistles are the primary social vocalization among the majority of Delphinidae species (Janik & Slater, 1998), they are comparatively rare among killer whales, though they also tend to occur more often in social contexts than in nonsocial contexts (Ford, 1987; Thomsen, Franck & Ford, 2001).

In the northeast Pacific, dedicated studies on killer whales began in the 1970s and revealed the presence of three distinct ecotypes in the region: fish-eating residents, mammal-eating transients (also known as Bigg’s), and the lesser-known offshores (Bigg, 1982; Bigg et al., 1990; Ford, Ellis & BalcombIII, 1996). Resident killer whales were so named for their habit of spending portions of each year in a smaller geographic region focusing on specific salmon runs. Transient killer whales were initially more rarely encountered and roamed more widely, though recently they have established more of a regular coastal presence and are increasingly referred to as Bigg’s killer whales in honor of the pioneering killer whale researcher Michael Bigg. Offshores, as their name suggests, are more pelagic and are only rarely encountered in coastal waters. They are known to eat a variety of fish species including sharks (Ford et al., 2011). These three sympatric ecotypes are not known to socially interact or interbreed with one another (Barrett-Lennard, 2000).

Each of these killer whale ecotypes has a unique vocal repertoire that in some cases can vary among sub-populations of that ecotype. For instance, two distinct populations of fish-eating killer whales are found in the coastal waters of the continental United States and British Columbia: the Northern and Southern Resident killer whales. The Northern Residents are found primarily off northern Vancouver Island and central BC while the Southern Residents range off Southern Vancouver Island and on the outer coasts of Washington, Oregon, and California. There is some geographic overlap in their ranges but the two populations do not actively associate and are acoustically distinct (Ford & Fisher, 1983; Ford, 1987).

The Northern Residents are made up of 13 pods divided into three acoustic clans, which are defined as a group sharing a set of discrete calls (Ford, 1991). Pods within a clan may share discrete calls, while pods from different clans do not. All three Southern Resident pods (J-Pod, K-Pod, and L-Pod) are considered part of a single acoustic clan, J-Clan. J-Clan discrete calls have been classified alphanumerically (Ford, 1987), with the letter “S” preceding the number to indicate that it is from a Southern Resident (S1, S2, etc.). All three pods share some calls in common, while other calls are produced by only a single pod.

As with pulsed calls, resident killer whales also emit both stereotyped and variable whistles. Four stereotyped whistles have been identified in J-Clan and follow a similar alphanumeric designation (SW1, SW2, etc.) as defined by Riesch, Ford & Thomsen (2006), while twelve stereotyped whistles have been identified among the three acoustic clans that make up the Northern Residents (Riesch, Ford & Thomsen, 2008). While discrete call production is dominated by stereotyped vocalizations with up to 85–95% of phonations falling into stereotyped categories (Ford & Fisher, 1983; Hoelzel & Osborne, 1986), 70% of whistles produced were found to be variable (Riesch, Ford & Thomsen, 2006).

Both discrete calls and whistles can also occur in sequences, some of them repetitive in nature with the same stereotypes occurring multiple times sequentially (Hoelzel & Osborne, 1986; Riesch, Ford & Thomsen, 2008). Whistle sequences have been analyzed in greater detail in Northern Residents than in Southern Residents, with 84% of whistles found to occur in sequences while only 16% of them occur as standalone whistles (Riesch, Ford & Thomsen, 2008). In addition to non-random transition patterns, Riesch, Ford & Thomsen (2008) also provided evidence for whistle elements described as “stammers”, partial whistles occurring at the beginning or end of a whistle sequence, and “bridge elements”, connecting pieces within whistle sequences. Unlike with discrete calls, the same whistle types were seen across the three acoustic clans in the Northern Resident community of killer whales.

While the Southern Resident killer whales, listed as endangered in both the US and Canada (COSEWIC, 2001; NOAA, 2005), are among the most well-studied cetaceans in the world, whistle studies for the Southern Residents have been scarce. Their whistles were first described by Ford & Fisher (1983) and Hoelzel & Osborne (1986). Later research showed that whistle types remain stable for at least 13 years (Ford, 1991; Riesch, Ford & Thomsen, 2006). Riesch, Ford & Thomsen (2006) was the last study to analyze the whistle repertoire of the Southern Residents using recordings from 1979–1982. The existence of stammers and bridge elements have not yet been investigated in Southern Residents, nor have whistle sequences been explicity studied.

The present study aims to further investigate the whistle repertoire of Southern Resident killer whales using the stereotypes and associated spectrograms presented by Riesch, Ford & Thomsen (2006) as the primary reference. By using recordings from 2006–2007 and 2015–2017, the continued occurrence of the four original stereotyped whistles was assessed across a much longer time period, and additional analysis was undertaken to identify any possible new stereotyped whistles. The goal is to characterize currently used whistle stereotypes among this endangered population in order to provide the groundwork for future studies on the behavioral context, pod-level repertoires, and function of whistle usage in resident killer whales. A further understanding of whistle usage in Southern Resident killer whales may, as with studies on discrete call dialects, provide insights into their behavior, culture, and the transmission of vocal traditions (Deecke, Ford & Spong, 2000).

## Materials & Methods

### Data collection

The data were collected in the waters around San Juan Island, Washington, USA (48°32′N, 123°05′W), primarily in Haro Strait, using multiple uncalibrated omnidirectional hydrophones and recorders between the months of May and September from 2006–2007 and 2015–2017. Shore-based recordings made at Lime Kiln Lighthouse on the west side of San Juan Island in 2006 and 2007 were made using a permanently mounted hydrophone at a depth of ∼8 m (Cetacean Research Technology C304) and recording to a PC computer using Sound Forge (©2003–2017 Sony Creative Software). Recordings were initiated when whales were within the visual range of the lighthouse, typically ∼800 m, and continued until all whales had passed beyond that distance. Additional recordings made at Lime Kiln in 2015–2017 used the same data collection parameters with a hydrophone (Reson TC4032) mounted at a 23 m depth and recorded to a PC computer with Audacity^®^ (©1999–2021 Audacity Team). Further recordings in 2015–2017 were made from a ∼5 m runabout boat, operated while following all federal and state vessel regulations and the regional Be Whale Wise whale-watching guidelines. The hydrophone (Cetacean Research Technology SQ26-H1) was deployed from the vessel to a depth of ∼4m and recordings were made with a Zoom H1n solid state digital recorder. All recordings were made at a sampling rate of 44.1 kHz with the exception of a subset of recordings from 2015 that were made with a sampling rate of up to 96 kHz. Pod(s) present during the recordings were confirmed with photo-identification images taken with DSLR cameras referenced against the Center for Whale Research photo ID guides (Center for Whale Research, 2019).

### Data analysis

Prior to starting the assessment of recordings, the authors agreed upon a standardized approach for selecting whistles of suitable quality for analysis, requiring whistle spectrograms to be clearly visible and non-overlapping with other phonations. Out of an initial test recording of more than 40 whistles independently assessed by the authors, they had 100% agreement on which whistles were of acceptable quality for analysis. All recordings were then screened for high-quality whistles for analysis by the lead author, who also performed the initial categorization (stereotyped or variable) and identification (SW1, SW2, etc.). In addition to the Riesch, Ford & Thomsen (2006) study, the (Ford, 1987) discrete call catalogue was also referenced for pulsed discrete call types that have a whistle component (*e.g.*, S19, S36, S42). Whistles that were found to match with one of these call components were not included in the analysis to avoid potential false whistles detections. It is possible that the whales make these whistle components independently from the pulsed component of the call, but this was not investigated in the present study.

A subset of 100 whistles representing variable whistles and multiple examples of all stereotypes identified by the initial observer were selected and subsequently categorized by five additional observers (“coders”) experienced with categorizing stereotyped killer whale discrete call vocalizations but with no prior experience identifying stereotyped whistles. All coders analyzed the same subset of whistles and were asked to sort the whistles based on their spectrographic shape and sound into either stereotyped categories of their own design or to identify them as “variable” with no other matches existing in the set. An inter-rater reliability (IRR) test was performed to assess the reliability of the whistle categorizations across multiple coders. Since a fully-crossed design was executed utilizing more than three coders with more than two potential categories, Light’s kappa was computed as suggested by Hallgren (2012). Light’s kappa was computed by deriving the arithmetic mean of Cohen’s kappa across all pairs of coders. This overall index of agreement is reported as Light’s kappa (Light, 1971).

**Figure 1:**
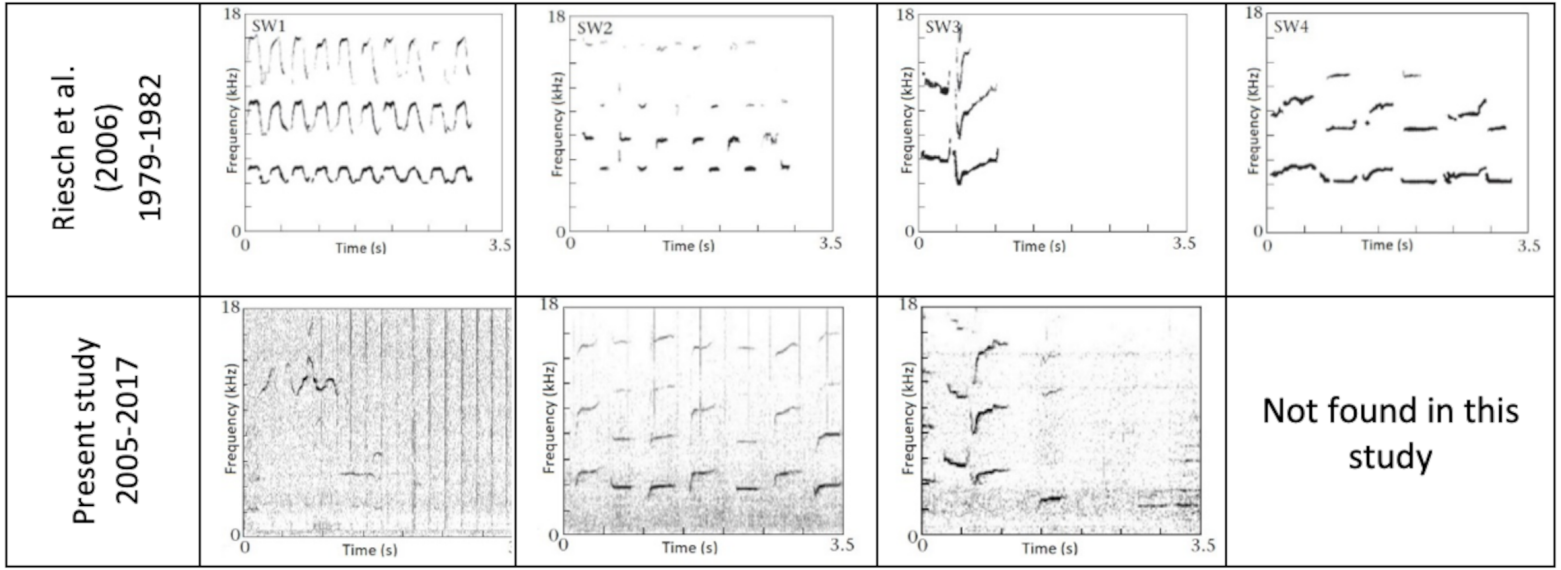
Spectrograms of the stereotyped whistles originally identified in (Riesch, Ford & Thomsen, 2006). Spectrograms (window type = Hann, DFT size = 512) of SW1, SW2, SW3, and SW4. Top row of spectrograms are reprinted from Animal Behaviour 71 (Riesch, Ford & Thomsen, 2006) “Stability and group specificity of stereotyped whistles in resident killer whales, Orcinus orca, off British Columbia” p.86 with permission from Elsevier. Bottom row of spectrograms are from recordings analyzed in the present study.

The initial analysis of recordings was done in Raven Pro software (Raven 1.6, Cornell Laboratory of Ornithology; sample rate = 44.1–96 kHz, frequency range 0 to 20–44 kHz, dynamic range = 48dB, window type = Hann, DFT size = 512). A spectrogram of each recording was visually scanned in a window showing 5.4 s of the recording at a time with a 0.5 s interval. When a sound looking visually similar to a whistle was detected (*i.e.,* a simple structure including only a few harmonics, as opposed to discrete calls), this section was aurally listened to in order to confirm the presence of a killer whale whistle. If a whistle was present, the observer manually categorized it into stereotyped or variable, with reference to the spectrograms of the four previously identified Southern Resident whistles identified by Riesch, Ford & Thomsen (2006): SW1, SW2, SW3, and SW4 (Fig. 1). The categorization of a new stereotyped whistle was considered when whistles could not be matched to SW1-SW4, had a similar spectrographic contour, and were found in at least three different recordings across at least two different years. The new whistle types were then numerically classified by the initial observer continuing the Riesch, Ford & Thomsen (2006) alphanumeric designations in the order that they were discovered (SW5, SW6, etc.).

Once whistles were identified, a selection box was drawn around the fundamental frequency in Raven Pro using the contour of the spectrogram as a guide. The selection table parameters saved with the annotation function were chosen following previous studies (Thomsen, Franck & Ford, 2001; Riesch, Ford & Thomsen, 2006) and included start and end time, low and high frequency, maximum frequency, bandwidth, and whistle duration. Also noted were whistle category (stereotyped or variable) and, if stereotyped, whistle ID. Data from each whistle were extracted from Raven, combined in an Excel spreadsheet with details about the recording including date and pod(s) present, and imported into R Studio IDE (R Studio Team, 2021). Data were analyzed with R 4.1.0 (R Core Team, 2021).

## Results

A total of 4,804 min (80 h) of recordings were analyzed from the years 2006–2007 and 2015–2017. Overall, 228 high quality whistles were extracted and analyzed, with a breakdown by year as follows: 86 from 2006, 53 from 2007, 39 from 2015, 42 from 2016, and 8 from 2017. A subset of 100 whistles were categorized by multiple coders. As reported in Riesch, Ford & Thomsen (2006), human observers generally agreed on the classification of stereotyped whistles. Light’s kappa was 0.66 (Cohen’s kappa range = 0.46–0.80), considered “substantial agreement” (Landis & Koch, 1977). Agreement extended to six of eight potential stereotyped categories identified by the initial coder. One previously identified stereotype (SW4, *n* = 3) and one potential new stereotype (*n* = 3) were rejected from further analysis due to both their low sample size and lack of support across all five additional coders.

Three of the four original stereotyped whistles identified in Riesch, Ford & Thomsen (2006) were confirmed in the present study: SW1 (*n* = 11), SW2 (*n* = 6), and SW3 (*n* = 16). In total, 34 of the 228 whistles analyzed (14.9%) in the present study were categorized into one of these three stereotypes, compared to 45 of the 152 (29.6%) whistles from 1979–1982 analyzed by Riesch, Ford & Thomsen (2006) falling into one of the four stereotypes. Parameters for the three stereotyped whistles identified in both studies were similar between the two time periods (Table 1). Welch’s *t*-tests showed no significant difference in bandwidths [*t* (4) = 0.11, *p* = 0.92, 95% CI [−1.50–1.62]]or durations [t(4) = −0.25, *p* = 0.81, 95% CI [−7.84–6.57]] between the two time periods.

**Table 1:** Parameters of SW1, SW2, and SW3 between two time periods. Comparison of whistle parameters (S.D.) from Riesch, Ford & Thomsen (2006) (shaded rows) and from the current study (white rows) for whistles SW1, SW2, and SW3. Measurements were taken on the fundamental frequency of each whistle using the selection box feature in Raven Pro. Maximum frequency is defined as the most powerful frequency that occurred within the selection. Bandwidth 90% is defined as the frequency range containing 90% of the whistle energy.

**Whistle ID**	**Duration** **(s)**	**Low frequency (kHz)**	**High frequency (kHz)**	**Bandwidth 90%** **(kHz)**	**Maximum frequency (kHz)**
SW1 (*n* = 17)	6.23 ± 2.78	*NA*	*NA*	1.67 ± 0.55	6.02 ± 0.37
SW1 (*n* = 11)	1.16 ± 0.42	7.28 ± 2.58	11.17 ± 2.63	2.67 ± 0.74	8.46 ± 2.87
SW2 (*n* = 14)	3.67 ± 2.15	*NA*	*NA*	2.81 ± 0.92	7.76 ± 1.31
SW2 (*n* = 6)	7.02 ± 3.89	3.26 ± 0.41	7.04 ± 3.19	3.11 ± 2.52	4.06 ± 0.37
SW3 (*n* = 8)	1.00 ± 0.18	*NA*	*NA*	3.09 ± 1.22	7.33 ± 1.22
SW3 (*n* = 17)	0.82 ± 0.16	4.07 ± 0.25	6.85 ± 1.45	1.97 ± 1.24	4.86 ± 0.64

**Figure 2:**
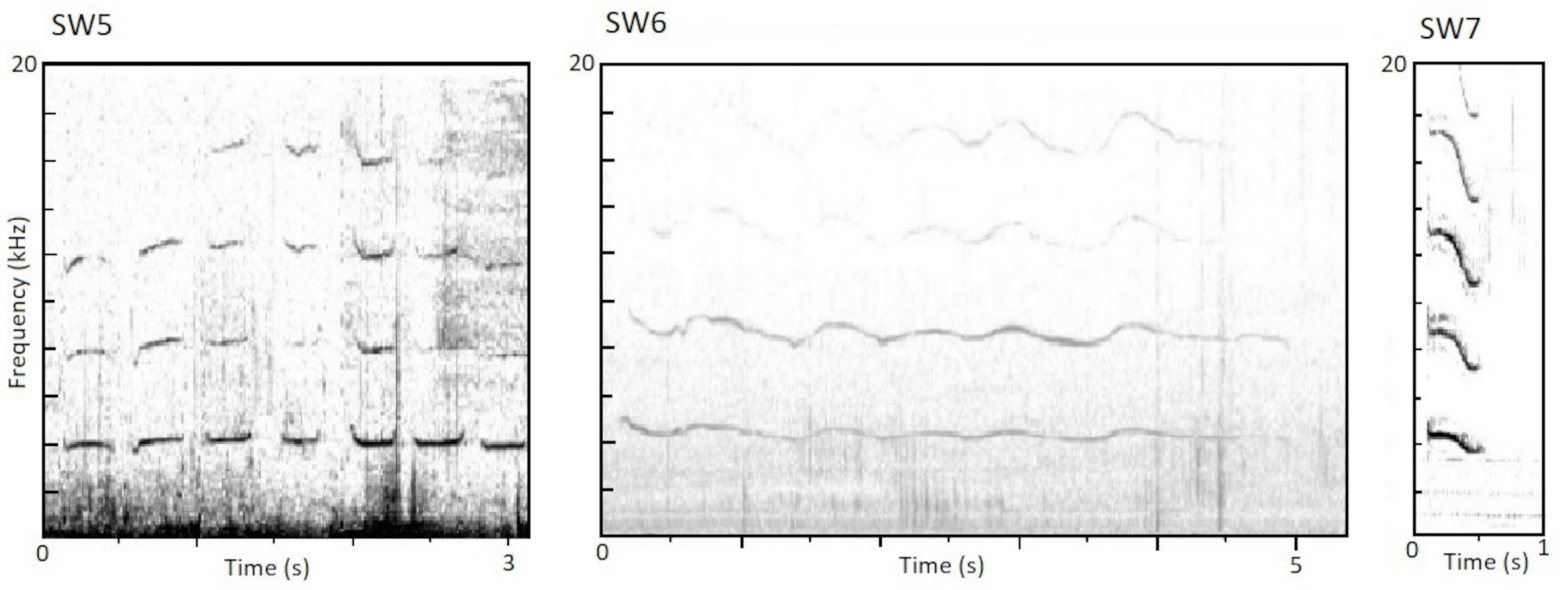
Spectrograms of the newly identified stereotyped whistles. Spectrograms of SW5, SW6, and SW7, newly identified in this study. (Window type = Hann, DFT size = 512).

In addition to three of the four stereotyped whistles described in Riesch, Ford & Thomsen (2006), three new stereotyped whistles were also found during the present study and designated SW5, SW6, and SW7. SW5 (*n* = 28) is similar to SW2 in that it is a series of repeating notes, though unlike SW2 the notes do not alternate back and forth between higher and lower frequencies but are identical in their characteristics. SW6 (*n* = 16) has the appearance on the spectrogram of being a “shallow wave”, with much more moderate frequency variation than SW1. SW7 (*n* = 45) was the most common new stereotype discovered and also had the strongest agreement among all coders with 97% of responses confirming the classifications made by the original coder. All three new stereotyped whistles were documented in 2006, 2007, 2015, and 2016; due to the much smaller sample size from 2017, whistle type usage was not assessed for this year. Figure 2 shows spectrograms of these newly identified stereotypes. Details of the parameters of the fundamental frequency of the new stereotypes are presented in Table 2. In total, 89 of the whistles analyzed (39.0%) fell into one of these three newly identified stereotype categories, while 122 of the 228 whistles analyzed (53.5%) were identifiable into one of the six total stereotyped categories.

**Table 2:** Whistle parameters for new stereotyped whistles SW5, SW6, and SW7. Parameters (S.D.) for the newly identified stereotyped whistles as well as the variable whistles measured in the current study. Measurements were taken on the fundamental frequency of each whistle using the selection box feature in Raven Pro. Maximum frequency is defined as the most powerful frequency that occurred within the selection. Bandwidth 90% is defined as the frequency range containing 90% of the whistle energy.

**Whistle ID**	**Duration** **(s)**	**Low frequency (kHz)**	**High frequency (kHz)**	**Bandwidth** **90%** **(kHz)**	**Maximum frequency (kHz)**
SW5 (*n* = 28)	4.38 ± 1.99	3.67 ± 0.81	5.55 ± 2.18	1.40 ± 1.84	4.35 ± 1.00
SW6 (*n* = 16)	1.69 ± 1.04	4.91 ± 1.69	6.22 ± 2.13	0.92 ± 0.60	5.41 ± 1.67
SW7 (*n* = 45)	0.33 ± 0.05	3.38 ± 0.21	5.96 ± 3.24	1.80 ± 2.33	4.09 ± 1.02
Variable (*n* = 105)	2.38 ± 2.18	4.44 ± 1.54	6.93 ± 2.73	2.09 ± 1.97	5.18 ± 2.13

In addition to strong observer agreement on the identification of the six stereotyped whistles, a comparison of the parameters between whistle types found significant differences in duration (ANOVA: *F*_5,117_ = 54.25, *p* < 0.001), low frequency (ANOVA: *F*_5,117_ = 27.63, *p* < 0.001), high frequency (ANOVA: *F*_5,117_ = 8.17, *p* < 0.001), and maximum frequency (ANOVA: *F*_5,117_ = 21.22, *p* < 0.001). Bandwidth was not significantly different between whistle types (ANOVA: *F*_5,117_ = 2.11, *p* = 0.069).

The recordings analyzed in this study varied between having single or multiple pods present, but utilizing the pod-specific recordings only, analysis was possible on 26 whistles from J-Pod, 28 from K-Pod, and 21 from L-Pod. Overall, a higher percentage of K-Pod (75%) and L-Pod (57%) whistles were stereotyped, while a higher percentage of J-Pod (62%) whistles were variable. When the number of stereotyped and variable whistles from each pod were compared to each other, there was a significant difference between the distribution of data across the three pods (chi-squared test, *χ*^2^ = 9.1, *df* = 2, *p* = 0.01). Of the six stereotyped whistles, no single pod produced all of them, though J-Pod was documented using five of the six (Table 3). K-Pod was confirmed to use two types and L-Pod four types. The new stereotype SW7 only occurred in recordings where K-Pod was present.

**Table 3:** Stereotyped whistle usage by pod. Whistle type usage by pod (shaded = present, white = absent) for recordings where only a single pod was present.

	SW1	SW2	SW3	SW5	SW6	SW7	Variable	**Total**
J	1	1	1	2	3		18	**26**
K					1	19	8	**28**
L	6	4	1	1			9	**21**

## Discussion

Although numerous studies have described pulsed discrete calls, the dominant social vocalizations of Southern Resident killer whales, few studies to date have investigated their usage of whistles. In comparing whistles from more recent recordings (2006–2007; 2015–2017) to the 1979–1982 recordings analyzed in Riesch, Ford & Thomsen (2006), the persistence of SW1, SW2, and SW3 was verified. This demonstrates that three of the four previously identified stereotyped whistles are stable across a time period of 38 years, more than three times longer than previously shown and establishing that stereotyped whistles are maintained over similar time periods as discrete calls. While there was no significant difference in the duration or bandwidth of the fundamental frequency of these whistles between the two time periods, the overall frequency range of whistles is still not well documented.

The previous study analyzed up to 18 kHz and the majority of recordings in this study measured up to 22 kHz, but it was anecdotally noted that in a subset of 2015 recordings that measured up to 48 kHz, Southern Resident whistle components could also be detected up to at least 43 kHz. This is a particularly interesting finding since ultrasonic whistles were detected in Atlantic killer whale populations, including some with fundamental frequencies up to 75 kHz, while ultrasonic whistle components were not detected in Northern Residents or Bigg’s killer whales in recordings that measured signals up to 48 kHz (Samara et al., 2010). This suggests that unlike these sympatric orca populations, Southern Residents utilize ultrasonic frequencies, and higher frequency hydrophones may be necessary to fully assess their acoustic signals.

Three new stereotyped whistles were also identified - SW5, SW6, and SW7 - which persevered across at least 10 years from 2006–2016 (not all types were detected in the more limited recordings from 2017). The high level of agreement among all six coders for these six stereotyped whistles was a particularly remarkable result since coders were not provided pre-existing stereotyped categories for reference but were asked to sort the whistles into stereotypes on their own. The identification of three new stereotyped whistles and the apparent loss of one historic stereotype also raises the question of how and how often whistles are added and dropped from the killer whale acoustic repertoire. While discrete call repertoires have proved generally stable over many decades, there is some evidence that discrete calls may be added (*e.g.*, Grebner et al., 2011) or dropped (*e.g.*, Wieland, Jones & Renn, 2010) from resident killer whale vocal traditions. It appears that stereotyped whistles may operate in the same fashion. There is a chance these new stereotyped whistles were undetected in the previous study, where these whistles were present but not categorized as stereotyped due to the smaller sample size or by simply being missed by the observers. However, the IRR tests from both studies show a high probability of observers detecting and matching stereotyped whistles (Riesch, Ford & Thomsen, 2006). The fact that two of the new whistles (SW5, *n* = 28; SW7, *n* = 45) were the most common whistle stereotypes documented in the present study is also evidence against the theory of failed detection, or at the very least demonstrates that their usage has dramatically changed since the previous time period.

There was a change in the composition of the Southern Resident pods between the two time periods due to births and deaths, but some members of all three pods were alive across the entire 38-year time period analyzed. Specifically, 8 whales in J-Pod, 6 whales in K-Pod, and 17 whales in L-Pod were born prior to 1982 and were still alive in 2005 (Center for Whale Research, 2021, pers. comm.). While there is no way to confirm which individual whales were emitting the whistles detected in either time period, given that some of the same whales were present for both studies, it is possible that individuals are shifting their acoustic repertoire over the course of their lifetime, as opposed to shifts occurring as a result of the change in membership of the population over time.

In the present study, 53.4% of the 228 whistles extracted for analysis were stereotyped whistles, nearly double the 30% of the 152 whistles previously found by Riesch, Ford & Thomsen (2006) from 1979–1982 recordings. Whether this represents an actual increase in stereotyped whistle usage is unknown due to the documentation of new whistle types in the present study. SW5-SW7 either went undetected in the previous study (an unlikely theory given the high coder agreement on identifying stereotypes across both studies) or are new whistle types to the Southern Resident repertoire. In either case, the newly identified stereotyped whistles are a likely explanation for the higher percentage of stereotyped whistles identified overall. Additionally, variable whistles may have been under-selected for inclusion in the analysis; with less recognizable spectrographic contours than stereotyped whistles, observer bias may be more likely to exclude them in recordings where spectrograms are impeded by background noise or other factors such as the positioning of the animal relative to the hydrophone. Finally, compared to the sample sizes of discrete call studies (on the order of several thousand calls), the whistle sample sizes in these two studies are relatively small due to the infrequent nature of whistle production. As additional recordings are made in the coming years, it will be interesting to note if additional whistle stereotypes emerge, and if some of the whistles designated as “variable” in these studies are just in fact more infrequently used stereotypes.

Another compounding factor in the identification and interpretation of stereotyped *vs.* variable whistles are the variants apparent within a single whistle type. Riesch, Ford & Thomsen (2006) also noted variation within categories, so just as with discrete calls, it appears there can be subtypes and/or “aberrant” whistles which are variations of a stereotype (Ford, 1987). It is difficult and somewhat arbitrary to assess at what point a stereotyped whistle has varied enough to instead be called a non-stereotyped variable whistle. SW2, a series of alternating higher and lower frequency notes, and SW5, a series of repeated notes at the same frequency, were particularly complicated in this regard. Two of the five additional coders lumped SW5 with SW2, while three coders agreed with the initial observer and sorted SW5 into its own discrete category. The fundamental frequencies of SW2 and SW5 did not show significant differences in bandwidth [*t* (6) = 1.57, *p* = 0.166, 95% CI [−932.22–4350.54]] or maximum frequency [*t* (23) = −1.19, *p* = 0.245, 95% CI [−787.76–211.79]], but were still designated unique stereotypes due to the distinct difference visible between alternating or repeating notes in the spectrograms and the strong agreement among four of the six observers for the distinction. For the present study, a whistle that did not conform to one of these two patterns was designated as variable, but variations on this repetitive whistle pattern were widely noted among the variable whistles. For example, a whistle might begin with a series of repetitive notes but then switch in the middle to alternating notes, or a whistle might be a repeated series of ascending notes rather than “flat” notes. The common usage of these long (>4 s) whistles with repetitive note components warrants further investigation in the future.

While the sample size of whistles analyzed from each pod on their own was not robust enough to conduct a statistical analysis, given the similarities between stereotyped whistles and stereotyped discrete calls, the possible existence of whistle dialects within an acoustic clan is an intriguing consideration. Interestingly, all whistle types were documented among more than one pod except SW7, which was also the most common whistle detected in the present study. SW7 dominated whistle usage in recordings of only K-Pod, making up 68% of K-Pod whistles analyzed, and among multi-pod recordings it only occurred in recordings where K-Pod was present, suggesting that this whistle type may be unique to them.

The fact that the whistle types used and the frequency of whistle usage varies by pod suggests that there may in fact be whistle repertoires among resident orcas alongside discrete call repertoires. Given the fact that whistles are stereotyped over long periods of time and are likely also transmitted *via* vocal learning, it is not surprising that this could be the case. There is also initial evidence that, just like among discrete call repertoires, one or two whistle types may dominate whistle usage of a particular pod, while other types may be present but used infrequently. If this proves to be true over time, whistles could eventually be used to contribute to pod-level identification of whale presence in passive acoustic monitoring efforts, which are of particular importance for the endangered Southern Residents in tracking their movements on the outer coast where visual observations are more infrequent. The existence of whistle repertoires would be especially interesting to investigate further given the previous finding that the three Northern Resident acoustic clans share stereotyped whistles (Riesch, Ford & Thomsen, 2006), suggesting that perhaps they were shared community-level rather than the clan-level of killer whale society. Whistles may thus function at different social levels between Northern and Southern Resident killer whales.

As in previous studies (Hoelzel & Osborne, 1986; Ford, 1991), observers noted Southern Resident whistles occurring in repetitive sequences in the present study. Some stereotyped whistles, particularly SW2 and SW5, are made up of notes repeated in highly variable fashion, so that the total duration of the whistle can vary from less than 4 to more than 13 s. Evidence of non-repetitive sequential relationships between different whistles was also noted. SW3 in particular was documented to occur at the beginning, middle, or end of other whistles, such as SW5. SW3 is similar in its structure and short duration to the Northern Resident bridges, however unlike the stammers and bridge elements noted in Riesch, Ford & Thomsen (2008), it was also documented as a stand-alone whistle. Looking at the association between Southern Resident whistle stereotypes as used in whistle sequences will be an interesting topic for future study, especially in comparison to the findings of whistle sequences in the Northern Residents.

While not analyzed in detail here, a cursory look was also taken at whistle usage relative to behavioral state. The vast majority of whistles detected occurred when the whales were traveling or socializing. A few whistles were documented during traditional foraging (defined here as the whales being spread out over a large area with non-directional heading relative to one another), while no whistles were heard during resting. These observations align with previous findings that the Southern Residents use whistles mainly in short-range interactions (Ford, 1989; Thomsen, Franck & Ford, 2001; Thomsen, Franck & Ford, 2002; Riesch, Ford & Thomsen, 2006; Riesch, Ford & Thomsen, 2008). It makes sense that whistles are more likely to be used when engaged in behavioral states where the whales are either in close proximity (socializing) or oriented in such a way that directional phonations like whistles are more likely to be received (such as when traveling, when whales all have the same heading). Whistles are less likely to be useful during foraging, when the whales are both spread out and oriented in various directions, especially since communication is less likely to be essential for resident killer whales who appear to hunt without cooperative foraging strategies (Ford & Ellis, 2014). However, it is not impossible that some whistles may be adapted for longer-range communication. Long-range signals are often stereotyped, spectrally simple, long in duration, and repetitive (Tyack & Miller, 2002), which are all characteristics of resident killer whale whistles. Traveling whales can be either in near or far proximity to one another, so the high usage of whistles while traveling (also noted by Riesch, Ford & Thomsen, 2006), may be an indication that they are adapted for this purpose as well. If further correlations of whistle usage and/or whistle types to particular behavior states are made, this could add another layer of analysis and understanding to passive acoustic monitoring efforts of the Southern Residents.

## Conclusions

Despite being such a well-studied species, analysis of whistle usage among killer whales is still relatively scarce. For the Southern Residents, even though not used nearly to the extent of their more common pulsed discrete calls, whistles appear to play a significant role in communication, demonstrated by the existence of at least 7 stereotyped whistles (3 of which are newly defined in the present study), some of which have shown stability for a time period of over 35 years. The possible evidence for whistle dialects at the pod level, the observations of whistles being associated with particular behavioral states, and the fact that they can potentially occur in non-random sequences all indicate that this is an area of killer whale behavior that warrants continued study. While there is a potential practical application of whistles being used alongside discrete calls to identify not only population but potentially pod presence through passive acoustic monitoring efforts, these findings are also intriguing as the function of whistles in killer whale communication and acoustic cultural traditions as a whole is considered further.

##  Supplemental Information

10.7717/peerj.12085/supp-1 Supplemental Information 1 Parameters of Analyzed Whistles Raw data containing parameters for the 228 whistles analyzed in this study, including year of recording, pod(s) present, duration, whistle type, and whistle ID. Also includes high frequency, low frequency, 90% bandwidth and max frequency amplitude for the fundamental frequency.

## Additional Information and Declarations

### Data Availability

The following information was supplied regarding data availability: The raw measurements of whistle parameters are available in the Supplementary Files.
